# *Helicobacter pylori* VacA Toxin/Subunit p34: Targeting of an Anion Channel to the Inner Mitochondrial Membrane

**DOI:** 10.1371/journal.ppat.1000878

**Published:** 2010-04-29

**Authors:** Grażyna Domańska, Christian Motz, Michael Meinecke, Anke Harsman, Panagiotis Papatheodorou, Boris Reljic, Elke A. Dian-Lothrop, Antoine Galmiche, Oliver Kepp, Lars Becker, Kathrin Günnewig, Richard Wagner, Joachim Rassow

**Affiliations:** 1 Institut für Physiologische Chemie, Ruhr-Universität Bochum, Bochum, Germany; 2 Institut für Biophysik, Universität Osnabrück, Osnabrück, Germany; 3 Laboratoire de Biochimie, INSERM ERI12, Hopital Nord, CHU Amiens Picardie, Amiens, France; 4 INSERM U848, Institute Gustave Roussy, Université Paris Sud, Villejuif, France; University of Illinois, United States of America

## Abstract

The vacuolating toxin VacA, released by *Helicobacter pylori*, is an important virulence factor in the pathogenesis of gastritis and gastroduodenal ulcers. VacA contains two subunits: The p58 subunit mediates entry into target cells, and the p34 subunit mediates targeting to mitochondria and is essential for toxicity. In this study we found that targeting to mitochondria is dependent on a unique signal sequence of 32 uncharged amino acid residues at the p34 N-terminus. Mitochondrial import of p34 is mediated by the import receptor Tom20 and the import channel of the outer membrane TOM complex, leading to insertion of p34 into the mitochondrial inner membrane. p34 assembles in homo-hexamers of extraordinary high stability. CD spectra of the purified protein indicate a content of >40% β-strands, similar to pore-forming β-barrel proteins. p34 forms an anion channel with a conductivity of about 12 pS in 1.5 M KCl buffer. Oligomerization and channel formation are independent both of the 32 uncharged N-terminal residues and of the p58 subunit of the toxin. The conductivity is efficiently blocked by 5-nitro-2-(3-phenylpropylamino)benzoic acid (NPPB), a reagent known to inhibit VacA-mediated apoptosis. We conclude that p34 essentially acts as a small pore-forming toxin, targeted to the mitochondrial inner membrane by a special hydrophobic N-terminal signal.

## Author Summary

VacA is a toxic protein produced by *Helicobacter pylori*, the bacteria that cause gastritis and ulcer diseases. p34, the toxic component of VacA, is known to damage mitochondria, defined cell organelles in the target cells. However, both the mechanism of mitochondrial targeting and the toxic activity inside the mitochondria are unclear. In this study, we show that p34 carries a unique targeting signal that is different from all targeting signatures that were previously identified in endogenous mitochondrial proteins. Eventually, p34 seems to act as an anion channel in the mitochondrial inner membrane and thus to destroy the balance of salt ions in the organelles.

## Introduction

*Helicobacter pylori* is a gram-negative bacterium infecting the human gastric mucosa, causing gastritis and peptic ulcer and, in some cases, gastric cancer [1]–[5]. One of the major virulence factors of the bacteria is the vacuolating toxin VacA, a protein of about 90 kDa [1], [6], [7]. VacA forms hexameric or heptameric flower-shaped oligomers [8]–[11]. These contain a central cavity and are able to form an ion channel [12]–[17]. The VacA toxin, as it is released by the bacteria, is a hetero-dimeric protein, comprising the subunits p58 and p34 that stay associated by non-covalent interactions [7], [18]–[20]. Similar to the subunits of A/B toxins, the two components have different functions in targeting and toxicity: The p58 subunit mediates binding to target cells. Following entry into host cells by endocytosis, the p34 subunit is essential to cause toxic effects [6], [21].

The p34 subunit is a polypeptide of 319 amino acid residues [22]. The first 32 residues are uncharged and required both for VacA insertion into the plasma membrane of host cells and for toxicity [7]. Inside the target cells, p34 can be imported into mitochondria [23], and mitochondria were shown to play an important role in the toxicity of the protein [24]–[29]. However, p34 does not reveal an obvious mitochondrial targeting signal, and nothing is known about the fate and the relevant molecular activities of p34 inside the mitochondria. p34 can dissipate the mitochondrial membrane potential, interfere with the mitochondrial energy metabolism and trigger apoptosis [23], [24], [30], but the mechanism is unclear. In this study we therefore asked: How is p34 imported into mitochondria? What is the mitochondrial targeting sequence? To which subcompartment is p34 targeted, and what is the activity of p34 inside the mitochondria? Combining biochemical and biophysical investigations, we found that the p34 subunit essentially acts as a small pore-forming toxin targeting the mitochondrial inner membrane by a peculiar import signal.

## Results

### Mitochondrial targeting of p34

**Figure 1:**
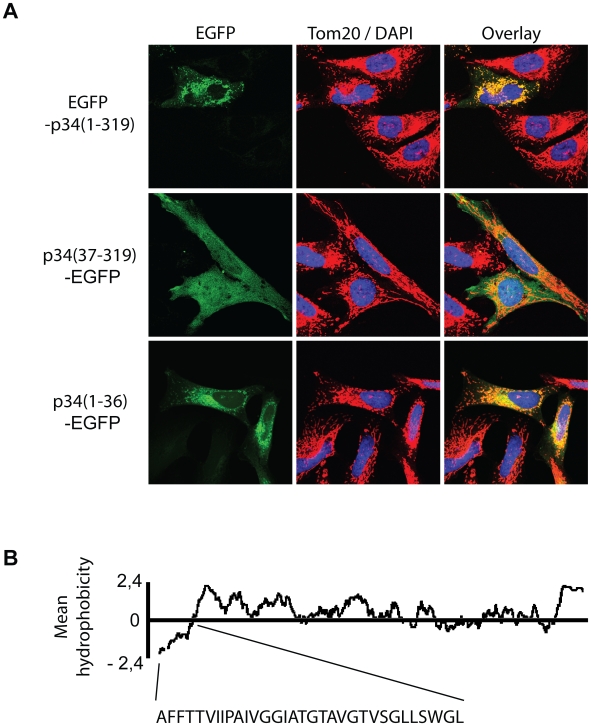
Distribution of p34 in intact cells. (**A**) Hybrid proteins containing an EGFP domain (enhanced green fluorescent protein) fused to the complete p34 (residues 1–319), or parts of p34 comprising residues 37–319 or 1–36, respectively, were expressed in HeLa cells (green fluorescence) and their distribution was monitored by confocal microscopy. About 10% of the cells were found to be transfected. Mitochondria were visualized with a Tom20-specific labelling (red colour; Tom20 is a subunit of the protein translocase of the mitochondrial outer membrane), the DNA of the cells was stained with DAPI (blue). The overlay (right panel) shows the co-localization between the EGFP-labelled proteins and Tom20 (yellow). (**B**) Hydrophobicity plot of p34(1–319) and sequence of the 32 N-terminal residues.

The p34 subunit of VacA is specifically imported into mitochondria, however it does not reveal an obvious mitochondrial targeting signal. To identify the segment that determines mitochondrial import, we designed hybrid proteins containing an EGFP moiety (enhanced green fluorescent protein) and investigated their distribution after expression in HeLa cells. Expression of p34-EGFP, a hybrid protein carrying the EGFP domain at the p34 C-terminus, causes cell death [23], however we confirmed that an EGFP-p34 protein containing the EGFP domain at the N-terminus was targeted to mitochondria keeping the transfected cells intact (Fig. 1A, upper panel). To determine the possible role of the hydrophobic p34 N-terminus in targeting, we tested a truncated construct comprising residues 37–319 of p34 fused to the EGFP domain. The construct stayed in the cytosol (Fig. 1A, middle panel.) Probably due to an affinity of the EGFP moiety [31], the construct partially co-localized with the nuclei of the cells. On the other hand, we found that the 36 N-terminal residues of p34 were sufficient for targeting of the EGFP domain to mitochondria (Fig. 1A, lower panel). Minor differences in the distribution of EGFP-p34(1–319) and p34(1–36)-EGFP suggest that the interactions of the N-terminal residues with the mitochondria may be facilitated by the authentic subunit. However, the N-terminal residues of p34 were essentially sufficient for targeting. The first 32 residues of p34, followed by a lysine in position 33, are non-charged or hydrophobic (Fig. 1B). They appear to represent a novel type of a mitochondrial targeting signal. The N-terminus of p34 is both essential and sufficient for mitochondrial targeting.

**Figure 2:**
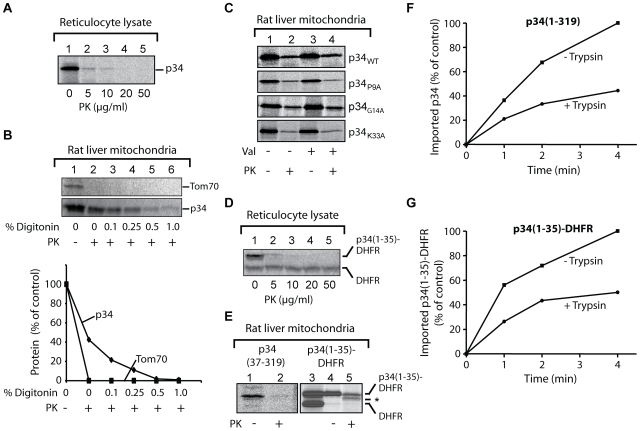
Import of p34 into isolated rat liver mitochondria. (**A**) Proteolysis of p34. Radiolabelled p34 was synthesized in reticulocyte lysate in the presence of ^35^S-methionine and incubated with increasing concentrations of proteinase K (PK) as indicated. The samples were incubated for 10 min at 0°C, proteolysis was stopped by addition of PMSF. Aliquots were analyzed by SDS-PAGE. The radiolabelled protein was visualized and the relative amounts were determined using a phosphoimager. At 10 µg PK/ml, about 1% of p34 was retained, no p34 was detected at 20 µg PK/ml. (**B**) Import of p34 into mitochondria and digitonin fractionation. ^35^S-labelled p34 and Tom70 (a subunit of 70 kDa of the protein translocase of the mitochondrial outer membrane) were synthesized in reticulocyte lysate and incubated with freshly isolated rat liver mitochondria for 10 min at 25°C. The mitochondria were reisolated by centrifugation and resuspended in 250 mM sucrose, 1 mM EDTA, 10 mM MOPS KOH, pH 7.2. As indicated, digitonin was added at increasing concentrations (up to 1% w/v), or proteinase K (PK) at a final concentration of 50 µg/ml, respectively. Proteolysis was stopped by addition of PMSF, and the mitochondria were collected by centrifugation. The proteins were separated by SDS-PAGE for subsequent analysis by digital autoradiography using a phosphoimager. (**C**) Import after exchange of residues in the p34 N-terminus. Reticulocyte lysate containing ^35^S-labelled p34 was incubated with mitochondria for 10 min at 25°C (upper panel). Parallel samples contained mutant versions of p34 (P9A, G14A, K33A). Samples 3 and 4 contained valinomycin to dissipate the membrane potential, samples 2 and 4 were subsequently incubated with proteinase K (PK, final conc. 25 µg/ml). The mitochondria were reisolated and the proteins were separated by SDS-PAGE. (**D**) Proteolysis of p34(1–35)-DHFR. The experiment was carried out as in (A). (**E**) Function of the p34 N-terminus. Left panel (lanes 1 and 2), incubation of N-terminally truncated p34 (residues 37-319) with mitochondria. Reticulocyte lysate containing the radiolabelled protein was incubated with isolated rat liver mitochondria as in (C). 25 µg/ml proteinase K were subsequently added to sample 2 to degrade the protein outside the mitochondria. Right panel (lanes 3–5), import of a hybrid protein comprising the 35 N-terminal residues of p34 fused to the entire dihydrofolate (DHFR) of the mouse. Lane 3, sample of reticulocyte lysate containing the radiolabelled hybrid protein p34(1–35)-DHFR and DHFR. Lanes 4 and 5, incubation of the lysate with mitochondria and reisolation of the mitochondria. Proteinase K was added to sample 5 (+PK). Asterix (*), degradation product of the hybrid protein. (**F**) Import of p34 into trypsin-pretreated mitochondria. Rat liver mitochondria were pretreated with trypsin (20 µg/ml) for 10 min at 0°C, proteolysis was stopped by addition of soybean trypsin inhibitor and the mitochondria were reisolated. ^35^S-labelled p34 was synthesized in reticulocyte lysate and incubated with the mitochondria at 25°C for different times as indicated. The trypsin pretreatment was omitted in parallel samples. The mitochondria were again reisolated and the proteins were separated by SDS-PAGE. The relative amounts of ^35^S-labelled p34 were determined using a phosphoimager. The highest value was set to 100% (control). (**G**) Import of p34(1–35)-DHFR into trypsin-pretreated mitochondria. The experiment was carried out as in (F).

To investigate the interactions of p34 with mitochondria in more detail, we used an *in vitro* assay (Fig. 2). For this purpose, we synthesized ^35^S-labelled p34 in reticulocyte lysate. Under these conditions, proteins are synthesized in the presence of the cytosolic proteins that determine the import-competence of mitochondrial proteins in mammalian cells [32]. Both hydrophilic and membrane proteins can subsequently be imported into isolated mitochondria. In the absence of mitochondria, 20 µg/ml of proteinase K (PK) were sufficient to completely hydrolyze p34 at 0°C within 10 min (Fig. 2A). We then incubated the ^35^S-labelled p34 with freshly isolated rat liver mitochondria at 25°C, the mitochondrial outer membrane protein Tom70 [33] was likewise synthesized in reticulocyte lysate and included as a control protein (Fig. 2B). After an incubation of 10 min, the mitochondria were reisolated by centrifugation and incubated in the presence of increasing concentrations of the detergent digitonin and proteinase K (PK, 50 µg/ml) at 0°C. Tom70 and p34 associated with the mitochondria (Fig. 2B, lane 1). In the presence of proteinase K (lane 2), Tom70 was rapidly degraded while about 40% of the p34 was retained, indicating that p34 was transported across the mitochondrial outer membrane. High concentrations of digitonin were required to allow the protease to get access to the p34 (Fig. 2B, lanes 3-6). In most of the experiments, about 20% of the p34 initially added to the samples was imported to a PK-protected location within 10 min.

Previous studies emphasized the relevance of distinct residues within the p34 N-terminus for VacA-induced cellular vacuolation [34], [35]. We separately exchanged three of these residues against alanine (P9A, G14A, K33A), but a substantial influence on the import efficiency was not observed (Fig. 2C). Moreover, the import was not blocked by the uncoupling reagent valinomycin, demonstrating that the mitochondrial membrane potential was not required in this reaction (Fig. 2C, lanes 2 vs. lanes 4).

To investigate the relevance of the N-terminal residues of p34, we used the construct p34(37–319) lacking the N-terminal sequence, or the hybrid protein p34(1–35)-DHFR comprising the N-terminal 35 residues of p34 linked to a complete DHFR (dihydrofolate reductase) domain. The p34 part of this construct was easily degraded by proteinase K, similar to the authentic p34 (Fig. 2D). The DHFR domain, however, was resistant even against high concentrations of the protease (Fig. 2D). Both constructs were synthesized in reticulocyte lysate and incubated with isolated rat liver mitochondria (Fig. 2E). The truncated version of p34 lacking the hydrophobic N-terminus was not imported (Fig. 2E, p34[37–319], lanes 1 and 2), in agreement with the observation that in intact cells, the p34(37–319)EGFP fusion protein did not co-localize with mitochondria (Fig. 1A). In a parallel assay, we tested the construct p34(1–35)-DHFR. The same samples also contained authentic DHFR. Upon incubation with mitochondria, p34(1–35)-DHFR efficiently associated with the mitochondria, while the DHFR was removed (Fig. 2E, lanes 3 vs. lane 4). The associated p34(1–35)-DHFR was partially protected against proteases, similar as the authentic p34 (in Fig. 2C), but a fraction of about 35% was degraded to a fragment of slightly smaller size (Fig. 2E, lane 5).

The observations show that the p34 N-terminus is necessary and sufficient for mitochondrial targeting of p34 both *in vitro* and *in vivo*. Since the p34(1–35)-DHFR fusion protein was only partially transported across the outer membrane, the p34 N-terminus might be a weaker import signal as compared to conventional positively charged mitochondrial targeting sequences. However, similar fragments as with p34(1–35)-DHFR were not observed with authentic p34. The p34 N-terminus thus appears to be sufficient for efficient targeting and import of the complete p34 subunit.

To act as a specific targeting signal, p34 should have the capability to interact with specific sites at the mitochondrial surface. These sites could be provided by mitochondrial outer membrane proteins. We pretreated rat liver mitochondria with trypsin at low concentrations, reisolated the mitochondria, and investigated if the import of p34 was affected. The rate of import was clearly reduced (Fig. 2F). We used the same mitochondria to import porin, a mitochondrial outer membrane protein, and found that the rate of import was similarly reduced (data not shown). Similar to endogenous mitochondrial proteins, import of p34 seems to be facilitated by proteins that are exposed at the mitochondrial surface. The effect was also observed with import of p34(1–35)-DHFR (Fig. 2G). In summary, the assays for import of p34 into rat liver mitochondria confirm that the p34 N-terminus can act as a mitochondrial targeting signal. The p34 N-terminus is sufficient to target specific recognition sites at the mitochondrial outer surface. The complete p34 is not only able to target mitochondria but to traverse the outer membrane and to accumulate inside the organelles.

**Figure 3:**
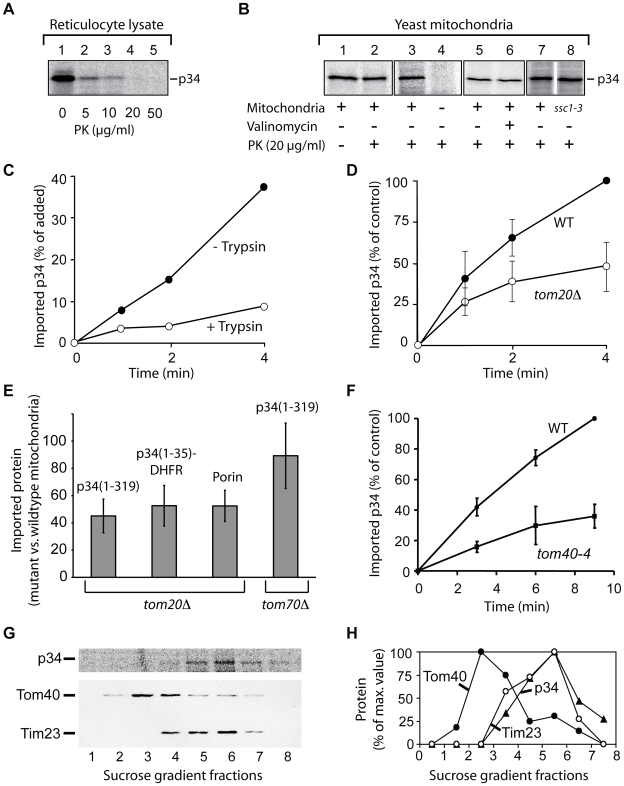
Import of p34 into isolated yeast mitochondria. (**A**) Protease-sensitivity of acid-pretreated p34. Reticulocyte lysate containg ^35^S-labelled p34 was pretreated with HCl at pH 5 for 5 min, diluted twentyfold into 1 mM EDTA, 10 mM MOPS/KOH pH 7.4, and incubated with proteinase K at increasing concentrations. (**B**) Import of p34 into mitochondria. p34 was imported as described in legend to Fig. 2, using yeast mitochondria instead of mammalian mitochondria. The organelles were isolated from the wild type strain PK82 (lanes 1–7) or from the mutant strain *ssc1-3* (lane 8). No mitochondria were added in the sample of lane 4. (**C**) Import of p34 in protease-pretreated yeast mitochondria. The mitochondria were incubated with trypsin (20 µg/ml) for 10 min at 0°C, proteolysis was stopped by addition of soybean trypsin inhibitor and the mitochondria were reisolated. ^35^S-labelled p34 was incubated with the mitochondria at 25°C for different times as indicated. (**D**) Import of p34 into mitochondria isolated from a strain lacking the outer membrane import receptor Tom20 (*tom20Δ*). Parallel samples contained mitochondria from the corresponding wildtype (WT). The standard deviation was calculated from 3 experiments, the highest value of each series was set to 100% (control). (**E**) Relative import efficiencies of p34(1–319), p34(1–35)-DHFR, and porin with mitochondria isolated from yeast strains lacking Tom20 or Tom70, respectively. The radiolabelled proteins were incubated with the mitochondria for 5 min at 25°C, reisolated, and treated with proteinase K. In parallel samples, the proteins were incubated with mitochondria isolated from the corresponding wildtype strains. The proteins were analyzed by SDS-PAGE and the relative amounts of the radiolabelled proteins were determined using the phosphoimager. The amounts detected in wildtype mitochondria were set to 100%. The standard deviations were calculated from 5 independent experiments. (**F**) Import of ^35^S-labelled p34 into mitochondria isolated from a yeast strain containing a defect in Tom40 (*tom40-4*). The highest value of each experiment was set to 100% (control), n = 3. (**G**) Import of radiolabelled p34 into mitochondria and preparation of outer and inner membrane vesicles. ^35^S-labelled p34 was imported into isolated yeast mitochondria, the mitochondria were reisolated and sonified. The membrane vesicles were layered on top of a sucrose step gradient and centrifuged for 16 h at 100.000 g. Fractions were collected for TCA-precipitation of proteins and subsequent analysis by SDS-PAGE, immuno blotting and visualization of radiolabelled p34 by digital autoradiography. Polyclonal antisera were used for labelling of Tom40 (outer membrane) and Tim23 (inner membrane), respectively. Fraction 1, upper part of the gradient (0.85 M sucrose); fraction 8, lower part of the gradient (1.6 M sucrose). Upper panel, radiolabelled p34 as visualized by digital autoradiography; lower panel, immuno blotting to visualize Tom40 and Tim23. (**H**) Quantification of the proteins shown in (G), the highest values were set to 100%.

To identify the structures that are targeted by p34, we used isolated yeast mitochondria as a model system (Fig. 3). To improve the import efficiency, we followed the observation that insertion of the VacA holotoxin into the plasma membrane is facilitated by an acid pre-treatment of the toxin [9], [13], [36]. We pre-incubated the lysate at pH 5, however, the import experiments were subsequently carried out at pH 7.2. The acid-pretreated p34 was completely degraded by proteinase K at a concentration of 20 µg/ml (Fig. 3A). After incubation with isolated yeast mitochondria, a fraction of p34 was protected against degradation (Fig. 3B, lanes 1 and 2), indicating that p34 was imported into the organelles. To exclude an unspecific aggregation of p34, we tested samples lacking mitochondria (Fig. 3B, lanes 3 and 4). Similar as with rat liver mitochondria, the import was independent of the mitochondrial membrane potential, as demonstrated by samples containing valinomycin (Fig. 3B, lanes 5 and 6). Import was also independent of mtHsp70 (encoded by *SSC1*; [37]), the major heat shock protein of 70 kDa in the mitochondrial matrix (Fig. 3B, lanes 7 and 8).

The import rate of p34 was significantly reduced if the yeast mitochondria were pretreated with trypsin, indicating that p34 similarly interacts with proteins at the surface of mitochondria from yeast and from mammalian cells (Fig. 3C). We took advantage of the availability of yeast mutants that show defined defects in the mitochondrial import machinery, and we isolated mitochondria from several of these strains. Tom20, a protein of the outer membrane, is the major receptor for import of endogenous proteins into mitochondria [32], [38]–[40]. Comparing the rate of import into mitochondria of a *tom20* deletion strain (*tom20Δ*; [41]) and the corresponding wildtype strain, we found that the import of p34 was significantly reduced (Fig. 3D).

The delay in import into mitochondria lacking Tom20 was also observed with p34(1–35)-DHFR (Fig. 3E). p34 and p34(1–35)-DHFR were similarly affected, and the results resembled data obtained with porin, which was again included as a mitochondrial standard protein (Fig. 3E). The experiments show that the N-terminal segment of p34 is able to recognize the import receptor Tom20. Interestingly, import of p34 into mitochondria from a *tom70* deletion mutant showed only minor effects (Fig. 3E, right column). Tom70 is a second import receptor besides Tom20 and involved in the uptake of a subset of mitochondrial proteins [32], [38]. p34 seems to specifically follow the Tom20 pathway for import.

For import into the inner compartments of mitochondria, proteins have to pass the general import pore that is mainly formed by Tom40 [32], [38], [40], [42], [43]. Using the mutant *tom40-4* [42], we found that Tom40 was clearly involved in the import of p34 (Fig. 3F). To enter mitochondria, p34 targets the same import pore as newly synthesized endogenous mitochondrial proteins.

Where is p34 localized after import into mitochondria? EGFP-p34 was previously detected by immuno gold labelling in the interior of HEp-2 cell mitochondria but the precise localization was unclear [23]. We imported radiolabelled p34 into isolated yeast mitochondria, disrupted the membranes by sonication, and separated the membrane vesicles by sucrose density centrifugation (Fig. 3G and H). The peak fractions of the outer membrane protein Tom40 and the inner membrane protein Tim23 were clearly separated, p34 was found to co-fractionate with Tim23. No significant amounts of p34 were detected in the outer membrane. In summary, we conclude that p34 targets the TOM complex in the outer membrane and subsequently accumulates in the mitochondrial inner membrane.

### Complex formation of p34

**Figure 4:**
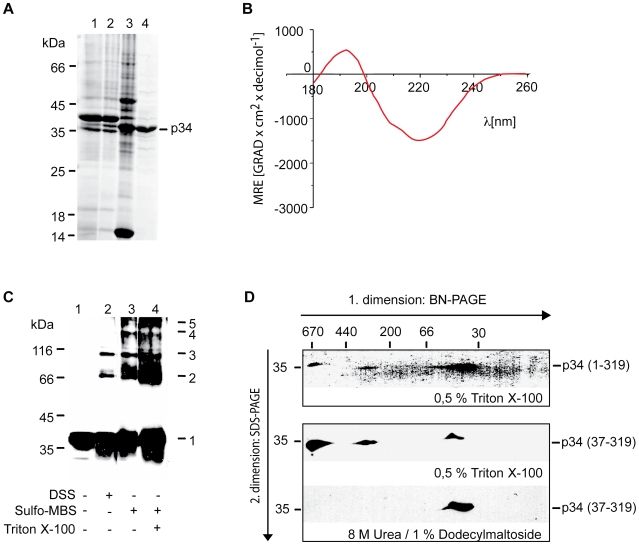
Complex formation of purified p34. (**A**) p34 was expressed in *Escherichia coli* and purified using conventional methods. Lane 1, inclusion bodies collected by centrifugation; lane 2, proteins dissolved in presence of 8 M urea and precipitated by ammonium sulfate (30% saturation); lane 3, eluate from Phenyl-Sepharose; lane 4, flow through from DEAE-Sephacel column. The proteins were separated by SDS-PAGE and the gel was stained by Coomassie. (**B**) CD spectra of p34. The purified protein was dissolved in 8 mM N-Decyl-β-D-Maltopyranosid, 10 mM KCl, 20 mM K_2_HPO_4_/KH_2_PO_4_, pH 7.0 and analyzed using a Jasco J-810 spectrapolarimeter. (**C**) Chemical cross-linking. Purified p34 was incubated with 50 µM DSS (lane 2) or 0.5 mM Sulfo-MBS (lanes 3 and 4) for 30 min at 0°C. The reaction was stopped by addition of 0.5 M Tris-HCl pH 7.4, the proteins were precipitated by TCA, separated by SDS-PAGE, transferred on nitrocellulose, and labelled using a polyclonal antiserum. (**D**) Analysis of p34 in BN-PAGE. Purified p34 was dissolved in 0.5% Triton X-100, 10% Glycerol, 50 mM NaCl, 0.1 mM EDTA, PMSF 1 mM, 20 mM Tris-HCl pH 7.0, and applied on a gel for BN-PAGE in the presence of 500 mM ε-aminocaproic acid (first dimension). A lane from the gel was excised and layered on top of a conventional SDS-PAGE for separation of the proteins under denaturing conditions (second dimension). The proteins were transferred on nitrocellulose and labelled using a polyclonal antiserum directed against p34 (upper panel). Truncated p34 comprising residues 37–319 was similarly analyzed (middle panel), or dissolved in the presence of 8 M urea and 1% dodecylmaltoside for separation in the first dimension (lower panel).

A previous study reported that after insertion of VacA into membranes, most parts of the p34 subunit are protected against proteases [44]. However, structure and function of p34 are still unclear. To obtain purified p34, we expressed p34 in *Escherichia coli* and isolated the protein by ammonium sulphate precipitation, hydrophobic chromatography and anion exchange chromatography (Fig. 4A). CD spectra of the purified protein indicated a high content of β-strands and a very low probability to form α-helices (Fig. 4B). The algorithm of the spectrapolarimeter calculated a content of 40–45% anti-parallel β-strands. The values are similar to results obtained with typical pore-forming β-barrel proteins [45]. A pore-forming activity of p34 is also indicated by the observation that an expression of p34 in HeLa cells entails a quick loss of the mitochondrial membrane potential [23]. Interestingly, using the program *TMB hunt* [46], we calculated a probability of p34 to form a β-barrel protein of 99%. Therefore we asked: Is the p34 subunit able to form a channel in the absence of the p58 subunit, and does the purified p34 oligomerize independently of p58?

We incubated purified p34 with the chemical cross-linking reagents DSS (Disucciminidylsuberate) or Sulfo-MBS (Sulfo-m-maleimidobenzoyl-N-hydroxysulfo-succinimide ester), respectively (Fig. 4C, lanes 1–3). With both reagents we observed a series of cross-linking products, indicating that p34 formed homo-oligomers containing several subunits. The same pattern of cross-linking products was observed in the presence and in the absence of Triton X-100 (Fig. 3C, lanes 3 and 4), suggesting that similar complexes assemble in the absence and in the presence of membranes. To determine the size of the complexes we carried out a blue native electrophoresis (BN-PAGE). In this system, the mobility of the proteins depends on their size, but also on their affinity for detergent molecules and for the Coomassie dye that is used to keep protein complexes in solution. In comparison to hydrophilic proteins, most membrane proteins show a reduced mobility [47]. Monomeric p34 showed a mobility similar to hydrophilic marker proteins of about 50 kDa (Fig. 4D, lower panel). In the presence of 500 mM ε-aminocaproic acid, two different complexes of native p34 were resolved, corresponding to an apparent molecular mass of about 330 or 650 kDa, suggesting that native p34 assembles in complexes of 6–7 or 12–14 monomers, respectively (Fig. 4, upper panel). Complexes of similar size were found if derivatives of p34 were tested lacking the N-terminal 36 amino acid residues (Fig. 4, middle panel). The uncharged N-terminus is obviously dispensable for complex formation.

**Figure 5:**
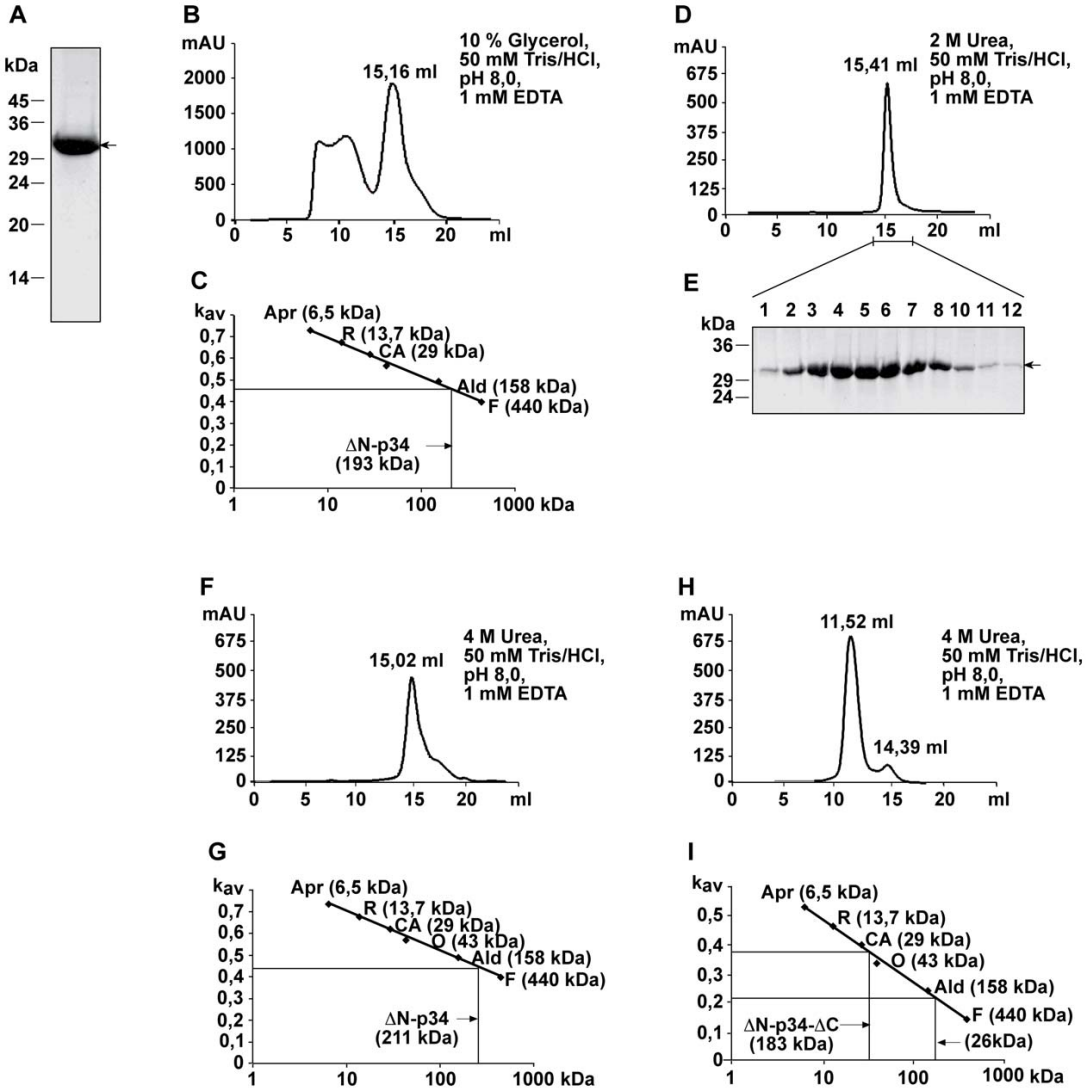
Analysis of p34 oligomers by size exclusion chromatography. (**A**) SDS-PAGE and Coomassie staining of 150 µg (His)_10_-p34 (comprising p34 residues 37–319) isolated by Ni-NTA chromatography. (**B**) (His)_10_-p34 was analyzed using the buffer as indicated. Oligomers were separated in a Superose 6 10/300 GL size exclusion column (diameter 10 mm, length 310 mm, flow rate 0.5 ml/min). (**C**) Determination of the apparent molecular mass of the (His)_10_-p34 oligomers in the 15.16 ml fraction of the elution profile shown in (B). Marker proteins Aprotinin (Apr, 6.5 kDa) from bovine lung, Ribonulease A (R, 13.7 kDa) from bovine pancreas, Carbonic Anhydrase (CA, 29 kDa) from bovine erythrocytes, Ovalbumin (O, 44 kDa) from hen egg, Aldolase (Ald, 158 kDa) from rabbit muscle and Ferritin (F, 440 kDa) from horse spleen. (**D**) Elution of (His)_10_-p34 in the same column in the presence of 2 M urea. (**E**) SDS-PAGE and Coomassie staining of (His)_10_-p34 peak fractions. (**F**) Elution of (His)_10_-p34 in the presence of 4 M urea. (**G**) Determination of the apparent molecular mass of the (His)_10_-p34 oligomers in the 15.02 ml fraction of the elution profile shown in (F). The marker proteins were the same as in (C). (**H**) Elution of (His)_10_-p34_ΔN/ΔC_ (comprising p34 residues 37–292) using the buffer as indicated. Oligomers were separated in a Superose 12 10/300 GL size exclusion column (diameter 10 mm, length 310 mm, flow rate 1.0 ml/min). (**I**) Determination of the apparent molecular mass of the (His)_10_-p34_ΔN/ΔC_ oligomers in the 11.52 ml and in the 14.39 fractions of the elution profile shown in (H).

To obtain higher amounts of the subunit, we purified a derivative of p34 containing a (His)_10_-tag instead of the hydrophobic N-terminus (p34 residues 37–319 connected to 10 histidine residues; Fig. 5A). Complex formation was then tested by size exclusion chromatography. In the presence of 10% glycerol, (His)_10_-p34 eluted in several fractions, partially corresponding to very high molecular mass, probably due to a tendency to form aggregates (Fig. 5B). From the K_av_ value of the most prominent peak fraction, we calculated a molecular mass of 193 kDa (Fig. 5C). Since (His)_10_-p34 is a protein of 32.2 kDa, we infer that the oligomers contained 6 subunits. To prevent aggregation of the protein, the chromatography was repeated in the presence of 2 M urea (Fig. 5D and E). p34 eluted under these conditions in a single peak corresponding to a molecular mass of the hexameric complex. The hexamers showed an impressive stability, even in the presence of 4 M urea most of the complexes were retained, while a smaller fraction started to dissociate (Fig. 5F and G).

Following the observation that the p34 N-terminus was not required for complex formation, we also tested a construct additionally lacking a segment of 28 residues at the p34 C-terminus (p34 residues 37–292 linked to a 10 histidine tag; Fig. 5H and I). In the presence of 4 M urea, most of the protein was stable. Only a small fraction dissociated and eluted in fractions corresponding to the molecular mass of the monomers (Fig. 5I).

### Electrophysiology of isolated p34

**Figure 6:**
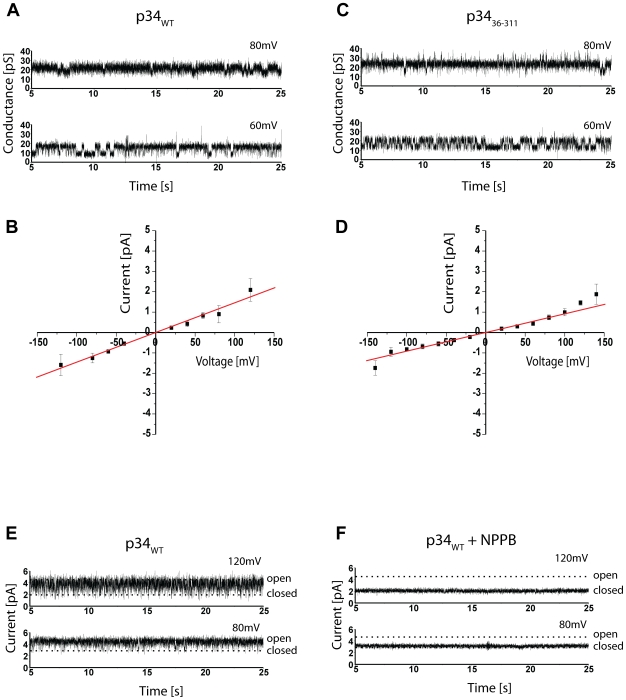
Electrophysiology of p34. (**A**) Current recordings of a bilayer containing purified p34 at indicated holding potential. Buffer conditions were symmetrical with 1.5 M KCl, 10 mM MOPS/Tris, pH 7.0 in *cis* and *trans* chamber. (**B**) Current-voltage relationship of p34. The conductance was calculated from the slope of the graph. (**C**) Current recordings of a bilayer containing p34(37–319) at holding potential as indicated. Buffer conditions were symmetrical with 1.5 M KCl, 10 mM Na-Acetat, pH 4.0 in *cis* and *trans* chamber. (**D**) Current-voltage relationship of p34(37–319). (**E**) Current recordings of a bilayer containing p34 at holding potential as indicated. The buffer conditions were the same as in (A). (**F**) Current recordings in the presence of 100 µM NPPB.

The possible pore formation of p34 was tested directly by incorporation of purified p34 in artificial membranes and subsequent electrophysiological characterization (Fig. 6). Current recordings confirmed a low but significant conductivity and a high dynamics in gating behaviour (Fig. 6A). A closer investigation of the current-voltage relationship revealed a conductance state of about 12 pS at 1.5 M KCl, pH 7.5 (Fig. 6B). Under asymmetrical buffer conditions the channels showed a clear preference for anions, with a P_Cl_/P_K_ value of 19 (data not shown). Similar electrophysiological characteristics were observed at pH 4 (not shown). p34 obviously acts as an anion channel of low conductivity. Based on the main conductance state, the diameter of the p34 ion channel can be calculated to be between 0.5 and 1.5 Å (assuming a cylindrical restriction zone of 1 nm with a five-fold higher resistance than the bulk medium). This dimension is sufficient to accommodate single chloride ions, but complex molecules such as organic acids should be excluded.

Interestingly we found similar values for the p34 construct lacking the hydrophobic N-terminus (p34_36–311_) (Fig. 6C and D). At 1.5 M KCl we determined a conductance state of about 10 pS (Fig. 6D), only slightly less as compared to the authentic p34. Similar data were also obtained with p34 containing exchanges of single residues within the N-terminus (P9A, G14A, K33A) or an N-terminal extension of 12 amino acids (s2 subtype, data not shown). It is therefore unlikely that the p34 N-terminus forms the ion channel.

The conductivity of some pore-forming toxins is modulated by ATP [48]. However, using a fluorescent-labelled ATP derivative, we did not obtain any evidence of ATP binding to p34 (not shown). Previous investigations demonstrated that the ion channel of VacA complexes can efficiently be blocked by NPPB (5-nitro-2-[3-phenylpropylamino] benzoic acid) [14]-[49]. Moreover, it was shown that the proapoptotic effect of VacA can be blocked by a preincubation of the target cells with NPPB [26]. A pretreatment of HeLa cells in the presence of 200 µM NPPB was demonstrated to prevent the cytochrome c release that is induced if purified VacA is added to the cells [30]. We added NPPB at a final concentration of 100 µM to our assay and found that it inhibited the conductance of the p34 channel completely (Fig. 6E and F).

## Discussion

In this study, we investigated targeting, mitochondrial import, the final location, and the function of the VacA p34 subunit of *Helicobacter pylori*. Because previous investigations on VacA were nearly exclusively carried out on the holotoxin [1], [7], [19], only little was known about the specific features of the toxic p34 subunit. Our data indicate that p34 acts as a pore-forming protein in the mitochondrial inner membrane.

It was previously shown that p34 targets mitochondria [23], but the targeting signal and the import pathway of the subunit remained enigmatic. We found that the 36 N-terminal residues of p34 are both necessary and sufficient for mitochondrial targeting. This observation is surprising because a similar mitochondrial targeting sequence was not described for any endogenous mitochondrial protein [32], [38], [40], [50], [51], or any other bacterial effector protein [52]. The 32 N-terminal residues of p34, shown in Fig. 1B, are uncharged or hydrophobic, the first charged residue of the sequence is the lysine in position 33. We found that the mitochondrial import receptor Tom20 is involved in the uptake of p34, in agreement with studies showing Tom20 to interact with hydrophobic residues of precursor proteins [53]. Subsequent transport of p34 across the mitochondrial outer membrane is mediated by the general import pore formed by Tom40.

In this case, the length and hydrophobicity of the p34 N-terminus seem to determine the specificity for import into mitochondria. Data on protein traffic in plant cells have already shown that the specificity for different membranes can be determined by the length of a hydrophobic segment of the sequence [54], [55]. Interestingly, it was demonstrated that a mitochondrial outer membrane protein can be changed to an inner membrane protein by an artificial increase in the mean hydrophobicity [56]. To our knowledge, the p34 subunit is the first example of an authentic protein that uses a hydrophobic N-terminus for targeting of the mitochondrial inner membrane. Membrane insertion of long hydrophobic peptides is membrane potential-independent [57], and in fact we found that p34 is efficiently imported both in the presence and in the absence of the mitochondrial membrane potential.

The p34 N-terminus is not only required for mitochondrial import but also for interactions of the VacA holotoxin with the plasma membrane during entry into the target cell [7]. The uncharged N-terminal sequence of the p34 subunit seems to confer both a general affinity for lipid bilayers, and specific targeting to mitochondria. The situation is reminiscent of the affinities of the outer membrane porin that similarly has a capability of spontaneous membrane insertion [58] but shows specific import into mitochondria depending on interactions with the mitochondrial TOM complex [42]. Some inner membrane proteins are first imported into the matrix compartment and subsequent insertion starts at the inner side of the membrane [32], however, we did not obtain any evidence of p34 import into the matrix. Because import of p34 is independent of the mitochondrial membrane potential and of matrix mtHsp70, it is more likely that p34 inserts at the outer surface of the inner membrane.

The oligomeric state of the complete VacA toxin was characterized in several studies [8]–[11]. We find that the p34 subunit alone is able to form highly stable complexes of about 200 kDa, corresponding to the molecular mass of 6 subunits. The formation of these hexamers is independent of membrane insertion, raising the possibility that soluble p34 monomers may pass the TOM complex, reassemble in the mitochondrial intermembrane space and insert into the inner membrane after complex formation. Assembly pathways of this type are well documented for bacterial small pore-forming toxins [59], [60]. A fraction of purified p34 formed oligomers of about 400 kDa in BN-PAGE (Fig. 4D), raising the possibility that p34 hexamers may form double donut-like structures under appropriate conditions. This assumption is supported by data on the complete VacA toxin that similarly showed a formation of 12mers [9], [10]. Previous data from a yeast two-hybrid assay had suggested that p58 may be essential for complex formation of p34 [18]. Our data on purified p34 demonstrate that this subunit is able to assemble autonomously, independently of the p58 component. The central part of p34 (residues 37–292) is sufficient for the formation of stable hexamers.

A crystal structure of the VacA p58 subunit was resolved [20], [61], but the structure of p34 has not yet been determined. Our CD spectra indicate that p34 has a content of >40% β-strands, probably in anti-parallel orientation. This value is similar to data reported for β-barrel proteins, although it does not exclude that the p34 β-strands may assemble in a different structure. The endogenous porin of human mitochondria, hVDAC1, shows a content of 32-37% β-strands, depending on the experimental conditions [45]. A high content of β-strands is also a feature of classical small pore-forming toxins, as exemplified by the α-hemolysin of *Staphylococcus aureus* (Gouaux, 1997). Similar to α-hemolysin, also p34 is able to form an anion channel of low conductance. The p34 complex seems to act essentially as a small pore-forming toxin targeting the mitochondrial inner membrane.

The pore-forming activity of the VacA holotoxin has been known for a long time [12]–[15], [49], [62], but the role of the two subunits had not been clarified. Surprisingly, we found that p34 is not only able to form a channel in the absence of the p58 subunit, but the conductivity of the p34 pore, about 12 pS in 1.5 M KCl buffer, is very similar to the values that were previously reported for the VacA holotoxin [12]. Both VacA [14], [62] and the purified p34 complex (this study) form an anion channel that can be blocked by the reagent NPPB, demonstrating that p34 is the essential pore-forming subunit of the toxin.

Several observations demonstrated that in the VacA holotoxin, the conductivity of the channel is dependent on the p34 N-terminus. For example, a formation of membrane channels was not detected with VacA containing an amino acid exchange P9A or G14A [16]. Following this observation, a model was proposed, suggesting that the p34 N-termini within the VacA toxin adopt an α-helical structure and associate to form the ion-conducting channel [17]. Remarkably, the first X-ray structures of pentameric ligand-gated ion channels recently confirmed that the central ion-conducting channel of these proteins is indeed formed by α-helices while other parts have a high content of β-strands [63]. However, other data already indicated that the p34 N-terminus might not be essential for pore formation. A mutant VacA protein lacking residues 6–27 oligomerized properly and showed a conductivity similar to the wildtype protein [34]. Strikingly, the current was detectable only after a much longer delay than when compared with the wildtype VacA [34]. Similar effects were described for the s2 subtype of the VacA toxin which is produced by some strains of *H. pylori*. s2 subtype VacA carries an additional peptide of 12 hydrophilic residues at the N-terminus [1], [7]. It forms membrane channels at a significantly reduced rate as compared to the abundant s1 type, but the channels exhibit similar anion selectivities [15]. Our experiments on the isolated p34 subunit indicate that the N-terminus is essential for mitochondrial targeting, but dispensable for assembly and for channel formation. We therefore regard it as unlikely that the p34 N-terminus is the channel-forming domain. Discrepancies between the data on the purified p34 complex and on the VacA holotoxin could be due to direct or indirect interactions between the N-terminus and the p58 subunit. Functional interactions with distant sites were reported for the N-terminus of *Staphylococcus aureus* α-hemolysin [64]. However, most effects of modifications in the p34 N-terminus, such as a reduced cell vacuolation [34] could be the consequence of an inhibition in membrane insertion rather than in conductivity.

Strains producing the s2 subtype are less virulent as compared to other strains [65]. We found that the s2 subtype p34 was imported into mitochondria with similar efficiency as the common s1 subtype, and it showed the same conductivity (data not shown). The observations confirm that the N-terminus is not relevant in determination of the channel properties. We assume that the reduced virulence of the corresponding *H. pylori* strains is due to effects in the pathway of the toxin from the plasma membrane to the mitochondria, in line with the observation that the N-terminus is primarily required for targeting in the cell.

It is currently unclear if p34 separates from p58 after uptake of VacA by target cells. However, our data show that both the VacA holotoxin and the p34 subunit carry the same mitochondrial targeting signal. Moreover, they indicate that both the VacA holotoxin and p34 are able to form hexamers that act as anion channels of very similar conductivity. What are the physiological consequences if these anion channels are formed in the mitochondrial inner membrane? It is difficult to asses the ionic equilibria inside mitochondria because the activities of the ions are largely determined by the extremely high protein content inside these organelles. A study on endosomal membranes suggested an interaction of VacA with pyruvate [66], raising the interesting question if the toxic activity of p34 complexes may depend on a depletion of the mitochondria from organic acids and thus from energy substrates. However, the permeability for pyruvate that was reported for the purified toxin was extremely low [49]. Our own data on the purified and reconstituted p34 indicate an inner diameter of the ion channel of only 0.5 to 1.5 Å. These dimensions correspond to the requirements of a single chloride ion and the ion channel should be unable to mediate the diffusion of complex molecules. We conclude that p34 essentially interferes with the homeostasis of inorganic anions inside the mitochondria.

An increasing number of studies already demonstrated a role of the VacA toxin as a mediator of apoptosis [23], [25], [26], [30], [67]–[69], but the relation of the VacA toxin to the mechanism of apoptosis remained unclear. Modifications of the inner mitochondrial membrane potential seem to be the first sign of a VacA activity in relation to cell death [27]. Remarkably, the reagent NPPB that we found to efficiently block the conductance of the p34 ion channel also inhibits VacA-dependent cytochrome c release and apoptosis [26], [30]. In the context of our data we suggest that (i) the anion conductivity described for the VacA holotoxin is due to the autonomous oligomerization and pore-formation of subunit p34, (ii) p34 contains an N-terminal signal for targeting to the mitochondrial inner membrane, deletion of the N-terminus abolishes transport to the mitochondrial target membrane. (iii) The p34 N-terminus is dispensable for pore-formation. (iv) Both pore formation and inner membrane targeting are essential features of p34 toxicity. The p34 subunit of the *Helicobacter pylori* VacA toxin may serve as a model system in further investigations on the pathological consequences of an anion channel in the mitochondrial inner membrane.

## Materials and Methods

### Human cell culture and fluorescence microscopy

HeLa cells were cultured in DMEM (Bio-Wittaker, Verviers, Belgium) with 5% fetal calf serum (Bio-Media, Boussens, France), penicillin/streptomycin and 2 mM L-glutamine (Gibco-BRL, Paisley, UK) at 37°C in an incubator with 5% CO_2_. For expression of EGFP-labelled proteins, the corresponding DNA fragments were cloned into the vectors pEGFP-C1 or pEGFP-N1, respectively (Clontech). The cells were transfected using Lipofectamine 2000 reagent (InvitroGen). Immunofluorescence labelling was performed following a conventional protocol. Briefly, 24 h after transfection, cells grown on glass coverslips were rinsed with PBS and fixed for 15 min with a 3.7% paraformaldehyde solution. After a 4 min permeabilization in PBS+0.1% Triton X-100, the coverslips were incubated in a 1∶100 dilution of a rabbit anti-Tom20 antibody (Santa-Cruz). A secondary antibody raised in donkey and coupled to Cy3 (Jackson Immunoresearch) was subsequently used, together with DAPI (Sigma). The coverslips were finally rinsed and mounted in Mowiol mounting medium (Calbiochem) prepared according to the manufacturer's instructions. Pictures were acquired on a TCS SP2 confocal microscope (Leica) equipped with a 63× HCX PL APO, NA = 1.40 objective (Leica), under oil immersion. Following acquisition, the images were combined using Photoshop software (Adobe).

### Construction of plasmids

Plasmids for expression in HeLa cells: The constructs encoding EGFP-p34(1–319) or p34(1–319)-EGFP, respectively, were described previously ([23]; the VacA sequence is available using the NCBI acc. no S72494.1 or GI∶619248). To obtain 34(37–319)-EGFP, the plasmid encoding p34(1–319)-EGFP was cut with XhoI and EcoRI within the p34 sequence, and the gap was closed using a synthetic DNA fragment encoding residues 37–58. For construction of p34(1–36)-EGFP, a synthetic DNA fragment encoding the N-terminal 36 amino acids of p34 was introduced in pEGFP-N1 via the XhoI and BamHI site. In both cases, the synthetic DNA fragments were obtained by hybridization of two complementary oligonucleotides, encoding the desired sequence together with an initiating start codon and containing the appropriate restriction site overhangs for subsequent cloning.

Plasmids for synthesis in reticulocyte lysate and for expression in *Escherichia coli*: For construction of p34(37–319), the plasmid pET28a-p34(1–319) was used as template [23]. This plasmid contains a NcoI site upstream of the p34 coding sequence. An additional NcoI site was introduced upstream of the codon of residue 37. By cutting with NcoI, the DNA segment corresponding to the N-terminal residues 1–36 was subsequently removed and the remaining plasmid was religated. For construction of p34(1–35)-DHFR, a BamHI site was introduced directly downstream of the codon of residue 35 in the plasmid pET28a-p34(1–319). The main part of the p34 coding sequence was subsequently removed by cutting with BamHI and HindIII and substituted by a DNA fragment encoding the entire DHFR (dihydrofolate reductase) of the mouse. (His)_10_-p34(37–319) was constructed by insertion of the p34 sequence encoding residues 37–319 into the plasmid pET10N (a modified pET19b plasmid; [70]). The DNA segment encoding p34(37–319) was obtained from pET28a-p34(1–319) by PCR, creating a NotI site upstream of the triplet encoding p34 residue 37, and subsequent cutting with NotI and XhoI. The DNA segment was ligated into pET10N downstream of the sequence for the dekahistidine tag. Due to the NotI site, the histidine residues of (His)_10_-p34(37–319) and the glutamic acid of position 37 are connected by a linker of three alanine residues. Essentially following the same procedure, a DNA insert encoding p34(37–292) was amplified from pET28a-p34(1–319) by PCR, creating a NotI site in front of the codon of p34 residue 37 and a XhoI site after the codon of residue 292.

### Import of proteins into isolated mitochondria

For import of radiolabelled proteins, rat liver mitochondria were used within 1 h after the isolation. The livers were obtained from animals of 80–120 g weight. Liver pieces were homogenized in buffer A (300 mM sucrose, 2 mM EGTA, 10 mM Tris-HCl pH 7.4) containing BSA (5 mg/ml, fatty acid free) and 1 mM PMSF using a Dounce homogenizer. Cell debris was removed by centrifugation (500 *g*, 10 min, 4°C). The supernatant was centrifuged at 12.000 *g* for 6 min to obtain a crude fraction of mitochondria. The mitochondria were subsequently resuspended in 12 ml buffer A, and Percoll (Sigma, P1644) was added to a final concentration of 5% (v/v). The mitochondria were pelleted by centrifugation at 17.000 *g* for 10 min, washed once in buffer A, and eventually resuspended in buffer A at a final concentration of 10 mg protein/ml. Yeast strains were grown in YPG medium (1% (w/v) yeast extract, 2% (w/v) bacto-peptone, pH 5.0, containing 3% (v/v) glycerol and mitochondria were subsequently isolated following standard procedures [71].

Radiolabelled proteins were imported into yeast and mammalian mitochondria following similar protocols [71]. The proteins were synthesized in rabbit reticulocyte lysate (TNT T7 Coupled Reticulocyte Lysate System, Promega, L4610) in the presence of ^35^S-labeled methionine (ICN Biomedical Research Products). For import into yeast mitochondria, the reticulocyte lysate containing p34 was preincubated with HCl (final concentration 30 mM) at pH 5–5.5 for 10 min at 25°C. (With most preparations of yeast mitochondria, the efficiency of p34 import without acid pretreatment was very low.) For import into mammalian mitochondria, the acid pretreatment was omitted. For protease-protection assays, the samples contained BSA buffer (3% [w/v] BSA, 80 mM KCl, 10 mM MOPS-KOH, pH 7.2), 2 µl reticulocyte lysate, 2 mM NADH, 1 mM ATP, 20 mM potassium phosphate and 30 µg (yeast) or 40 µg (rat liver) mitochondrial protein in a total volume of 100 µl. The import reactions were carried out at 25°C. The samples were subsequently cooled on ice and proteinase K was added at a final concentration of 25 µg/ml. Following an incubation for 10 min at 0°C, the protease was inactivated by 2 mM PMSF (phenylmethylsulfonyl fluoride) and an additional incubation for 5 min at 0°C. To dissipate the membrane potential, valinomycin (Sigma, V-0627) was used at a final concentration of 1 µM. Digitonin was used as described previously [72].

For preparation of membrane vesicles, mitochondria (2 mg protein in 200 µl SEM) were mixed with 200 µl 0.6 M Sorbitol, 20 mM HEPES-KOH pH 7.4 and incubated for 5 min at 0°C. 2.6 ml 0.5 M EDTA, 20 mM HEPES-KOH pH 7.4 and 100 mM PMSF were added for swelling of the mitochondria. Following an incubation for 30 min at 0°C, a mixture of protease inhibitors was added. Swelling of the mitochondria was stopped by the addition of sucrose to a final concentration of 1.8 M and an additional incubation for 10 min. Membrane vesicles were formed by sonication using a sonifier (Branson 250; duty cycle 70%, Output control 3). Each sample was treated with 3 cycles of each 30 sec. sonification and 15 sec., using a 3 mm Microtip (Heinemann, Schwäbisch Gmünd). The suspension of vesicles obtained by sonification was centrifuged for 10 min at 16.000 g to remove residual mitochondria. The vesicles were collected from the supernatant by centrifugation at 160.000 g (30 min., 4°C). The membranes were carefully resuspended in 400 µl 10 mM KCl, 5 mM HEPES-KOH pH 7.4 and 100 mM PMSF. The suspension was centrifuged for 10 min at 16.000 rpm×g to remove aggregates. The supernatant was applied on a step gradient of 0.85, 1.1, 1.35 and 1.6 M sucrose in 100 mM KCl, 5 mM HEPES-KOH pH 7.4 in a total volume of 11 ml, using Ultra-Clear centrifuge tubes (Beckman, 14×95 mm, No. 344060). The centrifugation was carried out for 16 h at 100.000×g using a SW41 rotor (Beckman, 30.000 rpm, 4°C). 1 ml fractions were collected for TCA precipitation and SDS-PAGE. The proteins were subsequently transferred on nitrocellulose and polyclonal antisera were used for labelling of marker proteins.

### Isolation of VacA subunit p34

p34 was expressed in *Escherichia coli*, strain C43(DE3), using the vector pET28a (Novagen). After an induction by 1 mM IPTG in a culture of 3000 ml for 3 h at 37°C, the cells were harvested and then opened using a french press. Inclusion bodies containing p34 were recovered by centrifugation and washed once in 100 mM urea, 1% (v/v) Triton X-100, 10 mM Tris/HCl pH 8.0, 0.1% (v/v) mercaptoethanol, and subsequently three times in 1 M urea, 10 mM Tris/HCl pH 8.0, 0.1% (v/v) mercaptoethanol. The inclusion bodies were eventually dissolved in 8 M urea, 100 mM Na_2_HPO_4_, 1 mM EDTA, 10 mM Tris/HCl pH 8 (PETurea, 5 ml/g cells), cell debris was removed by centrifugation. The supernatant was incubated for 30 min at 0°C with ammonium sulfate corresponding to a final concentration of 10% saturation. Precipitated proteins were removed and a fraction containing most of the p34 was precipitated from the solution by addition of ammonium sulfate at 30% saturation and 90 min incubation on ice. The precipitate was dissolved in PETurea buffer. The solution was diluted 1∶1 with 4 M urea, 1 M ammonium sulfate, 100 mM Na_2_HPO_4_, pH 8 and applied to a column containing 3 ml Phenyl-Sepharose (Amersham Pharmacia). The column was washed with fractions of decreasing concentrations of ammonium sulfate (500 mM, 300 mM) and p34 was eventually eluted with 4 M urea, 100 mM Na_2_HPO_4_, pH 8. The solution was dialyzed over night against 2 M urea, 1 mM EDTA, 50 mM Tris/HCl pH 8 (TEurea). The solution was then applied to a 3 ml DEAE-Sephacel column. p34 was easily eluted in subsequent washing steps using the TEurea buffer. Impurities were eventually eluted using TEurea buffer containing 1 M NaCl. Starting with 1 g *E. coli* cells, about 0.2 mg p34 was isolated. The truncated protein p34(37–319) and derivatives containing single amino acid exchanges were purified following the same protocol.

Derivatives of p34 (comprising residues 37–319 or 37–292) containing a (His)_10_-tag were isolated by Ni-NTA affinity chromatography. For expression, we used the *E. coli* strain C43. The expression was induced by addition of 1 mM IPTG following standard conditions and continued for 4 h at 37°C. The cells were opened using the sonifier (Branson 250; 2×1.30 min, 30% amplitude, 1.5 sec impulse, 0.5 pauses) and dissolved in 8 M urea, 1 mM EDTA, 50 mM Tris/HCl pH 8.0. Following a clarifying spin, the solution was applied to a 1 ml Ni-NTA column (Histrap, Amersham Pharmacia) using an *Äkta-Prime* system. (His)_10_-p34 eluted in a gradient at concentrations between 50 and 200 mM imidazole. The eluate was dialyzed over night against 4 M urea, 1 mM EDTA, 50 mM Tris/HCl pH 8 or 2 M urea, 1 mM EDTA, 50 mM Tris/HCl pH 8. The eluate was applied to a ‘Superose 6’ 10/300 or ‘Superose 12’ 10/300 column (Amersham Pharmacia). About 20 mg (His)_10_-p34 were obtained from 1 g *E. coli* cells. Samples of purified (His)_10_-p34 were used to immunize two rabbits and to obtain polyclonal antisera. The molecular mass of the eluted oligomers was determined using standard marker proteins (Amersham Pharmacia). The average elution position, Kav, was calculated using the equation Kav = (Ve - V_0_)*/*(Vt - V_0_), with Ve representing the elution volume, V_0_ the void volume, and Vt the total column volume.

### Cross-linking and BN-PAGE

For cross-linking, 30 µg p34 were dissolved in 0.5 ml 2 M urea, 1 mM EDTA, 10 mM MOPS, pH 7.2. DSS (Disucciminidylsuberate, Pierce Biotechnology Inc.) was used at a final concentration of 50 µM, Sulfo-MBS (Sulfo-m-maleimidobenzoyl-N-hydroxysulfo-succinimide ester, Pierce) was added at a final concentration of 0.5 mM as described previously [72]. Blue native electrophoresis (BN-PAGE) was carried out according to published procedures [47], [72]. Complexes of purified p34 (30 µg/lane), dissolved in 0.5% Triton X-100, 10% Glycerol, 50 mM NaCl. 0.1 mM EDTA, PMSF 1 mM, 20 mM Tris-HCl pH 7.0, were separated in gels containing 500 mM ε-aminocaproic acid (EACA).

### Electrophysiology

Electrophysiological characterization of p34 was carried out using the planar lipid bilayer technique as detailed in ref. [73]. Briefly, purified urea solubilised p34 was applied directly below the bilayer in the *cis* chamber. An acid pretreatment of the protein was omitted. Buffer conditions were symmetrical with 1.5 M KCl, 10 mM Mops-Tris (pH 7.0) in the *cis*/*trans* compartment or 1.5 M KCl, 10 mM Na-Acetat (pH 4.0) in the *cis*/*trans* compartment. Two Ag/AgCl electrodes covered by 2 M KCl-agar bridges were inserted into each chamber with the *trans* chamber electrode connected to the headstage (CV-5-1GU) of a Geneclamp 500 current amplifier (Axon Instruments) and thus was the reference for reported membrane potentials. A solution of purified azolectin (60 mg/ml; Sigma type IV-S) in n-decan (purity >99%, Sigma) was used to generate the planar lipid bilayers. Current recordings were carried out using a Digidata 1200 A/D converter. Data analysis was performed by self written Windows-based SCIP (single-channel investigation program) in combination with Origin 7.0 (Microcal Software). Current recordings were performed at a sampling interval of 0.1 ms, filtered with a low-pass-filter at 2 kHz.

### Circular dichroism-spectroscopy

Purified p34 was dialyzed against 8 mM N-Decyl-β-D-Maltopyranosid, 10 mM KCl, 20 mM K_2_HPO_4_/KH_2_PO_4_, pH 7.0. CD-spectroscopy and calculation of the secondary structure of p34 was performed as described in ref. [74]. Briefly, CD spectra were recorded using a Jasco J-810 spectrapolarimeter. All measurements were carried out in a quartz cuvette with an optical path length of 0.01 cm at room temperature. The scans (n = 16) were averaged to improve the signal/noise ratio. Blank buffer spectra were collected and subtracted from the sample spectra.
